# Decreased Frequencies of Circulating CD4^**+**^ T Follicular Helper Cells Associated with Diminished Plasma IL-21 in Active Pulmonary Tuberculosis

**DOI:** 10.1371/journal.pone.0111098

**Published:** 2014-10-24

**Authors:** Nathella Pavan Kumar, Rathinam Sridhar, Luke E. Hanna, Vaithilingam V. Banurekha, Thomas B. Nutman, Subash Babu

**Affiliations:** 1 National Institutes of Health–International Center for Excellence in Research, Chennai, India; 2 National Institute for Research in Tuberculosis, Chennai, India; 3 Government Stanley Medical Hospital, Chennai, India; 4 Laboratory of Parasitic Diseases, National Institutes of Allergy and Infectious Diseases, National Institutes of Health, Bethesda, Maryland, United States of America; University Medical Center Freiburg, Germany

## Abstract

**Background:**

Circulating T follicular helper (Tfh) cells represent a distinct subset of CD4^+^ T cells and are important in immunity to infections. Although they have been shown to play a role in experimental models of tuberculosis infection, their role in human tuberculosis remains unexplored.

**Aims/Methodology:**

To determine the distribution of circulating Tfh cells in human TB, we measured the frequencies of Tfh cells *ex* *vivo* and following TB - antigen or polyclonal stimulation in pulmonary TB (PTB; n = 30) and latent TB (LTB; n = 20) individuals, using the markers CXCR5, PD-1 and ICOS.

**Results:**

We found that both *ex* *vivo* and TB - antigen induced frequencies of Tfh cell subsets was significantly lower in PTB compared to LTB individuals. Similarly, antigen induced frequencies of Tfh cells expressing IL-21 was also significantly lower in PTB individuals and this was reflected in diminished circulating levels of IL-21 and IFNγ. This was not accompanied by diminished frequencies of activated or memory B cell subsets. Finally, the diminution in frequency of Tfh cells in PTB individuals was dependent on IL-10, CTLA-4 and PD-L1 *in* *vitro*.

**Conclusions:**

Thus, PTB is characterized by adiminution in the frequency of Tfh cell subsets.

## Introduction

Exposure to *Mycobacterium tuberculosis* (Mtb) can result in a variety of outcomes, including the absence of any clinical or laboratory evidence of infection, latent infection without active disease, active pulmonary disease or active extra-pulmonary disease [1]. Although 2 billion people worldwide are infected with Mtb, only 5–10% of these individuals develop active disease, and the mechanisms by which most individuals resist development of active disease are still not clear [1]. However, while by definition, individuals developing active TB exhibit a compromise in their ability to mount a protective immune response against MTB, the exact nature of this protective immune response needs to be determined. A wide range of specific and non-specific host immune responses are thought to contribute to the differential outcomes of infection and disease, although there is no unifying hypothesis to explain the differences seen [2].

Circulating Tfh cells are peripheral counterparts of conventional Tfh cells, that are predominantly located in secondary lymphoid tissues [3], [4]. Conventional Tfh cells are CD4^+^ T cells that express the chemokine receptor CXCR5, co-stimulatory molecules such as ICOS, PD-1, the transcription factor Bcl-6 and the cytokine, IL-21 [3], [4]. Circulating Tfh cells similarly express CXCR5, PD-1, ICOS but do not express Bcl-6 [5]. In addition, although some studies have defined circulating human Tfh cells as all CD4^+^ T cells expressing CXCR5^+^ only, other studies have suggested that CD4^+^ CXCR5^+^ T cells can be further divided into those that are PD-1^+^, ICOS^+^, and/or IL-21^+^ [6]. It is unclear whether expression of PD1, ICOS or IL-21 defines different subpopulations of Tfh cells [6]. Nevertheless, these cells are known to promote the differentiation of memory (but not naïve) B cells to plasma cells [5]. Dysregulated activity of conventional and circulating Tfh cells have been found to contribute to auto-immune or immune-deficiency states in several models of human disease [4], [7]. In addition, circulating Tfh cells have been shown to be biomarkers of effective humoral immunity in vaccination and infectious disease studies [8], [9], [10]. Finally, conventional Tfh (CD4^+^CCR5^+^) cells have been shown to mediate protective immunity against tuberculosis [11]. Thus, while the requirement for Tfh cells in animal models of TB infection is well-defined, the role of circulating Tfh cells in human TB infection and disease has not been explored.

To study the distribution of Tfh cells in TB infections, we examined Mtb antigen-specific induction Tfh cells subsets (defined as CD4^+^ CXCR5^+^ PD-1^+^ ICOS^−^ or CD4^+^ CXCR5^+^ PD-1^−^ ICOS^+^ or CD4^+^ CXCR5^+^ PD-1^+^ ICOS^+^) in PTB and LTB individuals in an area highly endemic for tuberculosis. We observed that active PTB was characterized by diminished frequencies of Tfh cells ex vivo and in response to TB antigens and by diminished frequencies Tfh cells producing IL-21, a finding that was reflected in circulating plasma levels of IL-21. IL-10, CTLA-4 and PD-L1 each appear to play a role in the Tfh homestasis as well. Our data therefore suggest that central defects in Tfh subset differentiation and/or function is a feature of active pulmonary tuberculous disease.

## Materials and Methods

### Ethics statement

All individuals were examined as part of a clinical research protocol approved by Institutional Review Board of the National Institute for Research in Tuberculosis, and informed written consent was obtained from all participants.

### Study population

We studied a group of 50 individuals; 30 with pulmonary TB (PTB) and 20 individuals with latent TB (LTB). Among the 30 individuals with PTB, 19 of these were also used for antibody blocking studies. Individuals with PTB were diagnosed sputum smear and culture positivity. Individuals with LTB were diagnosed on the basis of being positive in the Quantiferon-TB Gold in Tube (Cellestis) assay but having an absence of pulmonary symptoms concurrent with a normal chest radiograph. All subjects had been bacillus Calmette-Guerin (BCG) vaccinated at birth. All the individuals were HIV negative and blood was collected prior to commencement of anti-TB treatment.

### Antigens

Mycobacterial antigens - PPD (Statens Serum Institute, Copenhagen, Denmark), ESAT-6 and CFP–10 (both from NIAID TB antigen repository at BEI resources) were used as antigenic stimuli, and anti-CD3 antibody was used as positive control. Final concentrations were 10 µg/ml for PPD, ESAT-6 and CFP-10 and 5 µg/ml for anti-CD3. For antibody ELISA, PPD and MTB whole cell lysate (WCL) were used as the antigens.

### *Ex vivo* analysis

B cell phenotyping was performed using antibodies directed against CD 45, CD19, CD27, CD21, CD20 and CD10. Naïve cells were classified as CD45^+^ CD19^+^ CD21^+^ CD27^−^; classical memory B cells as CD45^+^ CD19^+^ CD21^+^ CD27^+^; activated memory B cells as CD45^+^ CD19^+^ CD21^−^ CD27^+^; atypical memory B cells as CD45^+^ CD19^+^ CD21^−^ CD27^−^; immature B cells as CD45^+^ CD19^+^ CD21^+^ CD10^+^; plasma cells as CD45^+^ CD19^+^ CD21^−^ CD20^−^.

### *In vitro* culture

Whole blood cell cultures were performed to determine the in vitro responses to antigens. Briefly, whole blood was diluted 1∶1 with RPMI1640 medium supplemented with penicillin/streptomycin (100 U/100 µg/mL), L-glutamine (2 mM), and HEPES (10 mM) and distributed in 12-well tissue culture plates. The cultures were then stimulated with ESAT-6, CFP-10, or anti-CD3 or with medium alone in the presence of CD49d/CD28 at 37°C for 6 hours. Brefeldin A (10 µg/mL) was added after 2 hours. After 6 hours, centrifugation, washing, and red blood cell lysis was performed. The cells were fixed using cytofix/cytoperm buffer and cryopreserved at −80°C. For neutralization experiments, whole blood was cultured in the presence of anti-IL-10 (5 µg/ml), anti-TGFβ (5 µg/ml) anti-PD-L1 (5µg/ml) and anti CLTA-4 (5 µg/ml) or isotype control antibody (5 µg/ml) at 37°C for 6 hours for 1 h following which PPD was added and Brefeldin A (10 µg/mL) was added after 1 hour. The cells were then cultured for a further 16 h.

### Intracellular cytokine staining

The cells were thawed, washed with cold PBS and permeabilized with 1x permeablization buffer. Cells were then stained with surface and intracellular antibodies together and incubated overnight for 4°C. Antibodies directed against CD3, CD4, CXCR5, IL-21, PD-1 and ICOS were used. Eight-color flow cytometry was performed on a FACSCanto II flow cytometer with FACSDiva software, version 6 (Becton Dickinson). The lymphocyte gating was set by forward and side scatter, and 100,000 lymphocytes events were acquired. Data were collected and analyzed using Flow Jo software (TreeStar). All data are depicted as the frequency of CD3^+^CD4^+^CXCR5^+^ cells expressing ICOS, PD-1 and IL-21. Baseline values following stimulation with medium are depicted as baseline frequency, while frequencies following stimulation with antigens are depicted as net frequencies (with baseline values subtracted).

### Enzyme-linked immunosorbent assay (ELISA)

Plasma IFNγ, IL–4 and IL-17A levels were measured, using kits from R&D Systems and IL-21 were measured by ELISA (eBioscience). The lowest detection limit for the various cytokines were: IFNγ − 2.14 pg/ml, IL-4 –0.3 pg/ml, IL-17A −2.57 pg/ml and IL-21 –2.2 pg/ml.

### Statistical analysis

Geometric mean was used as the measure of central tendency. Comparisons were made using either the Kruskal-Wallis test with Dunn’s multiple comparisons (unpaired comparisons) or the Wilcoxon signed rank test (paired comparisons). All statistics were performed using GraphPad Prism version 5 for Windows (GraphPad Software, Inc.).

## Results

### PD-1 and ICOS mark Tfh cells capable of responding to TB antigens in PTB

**Figure 1:**
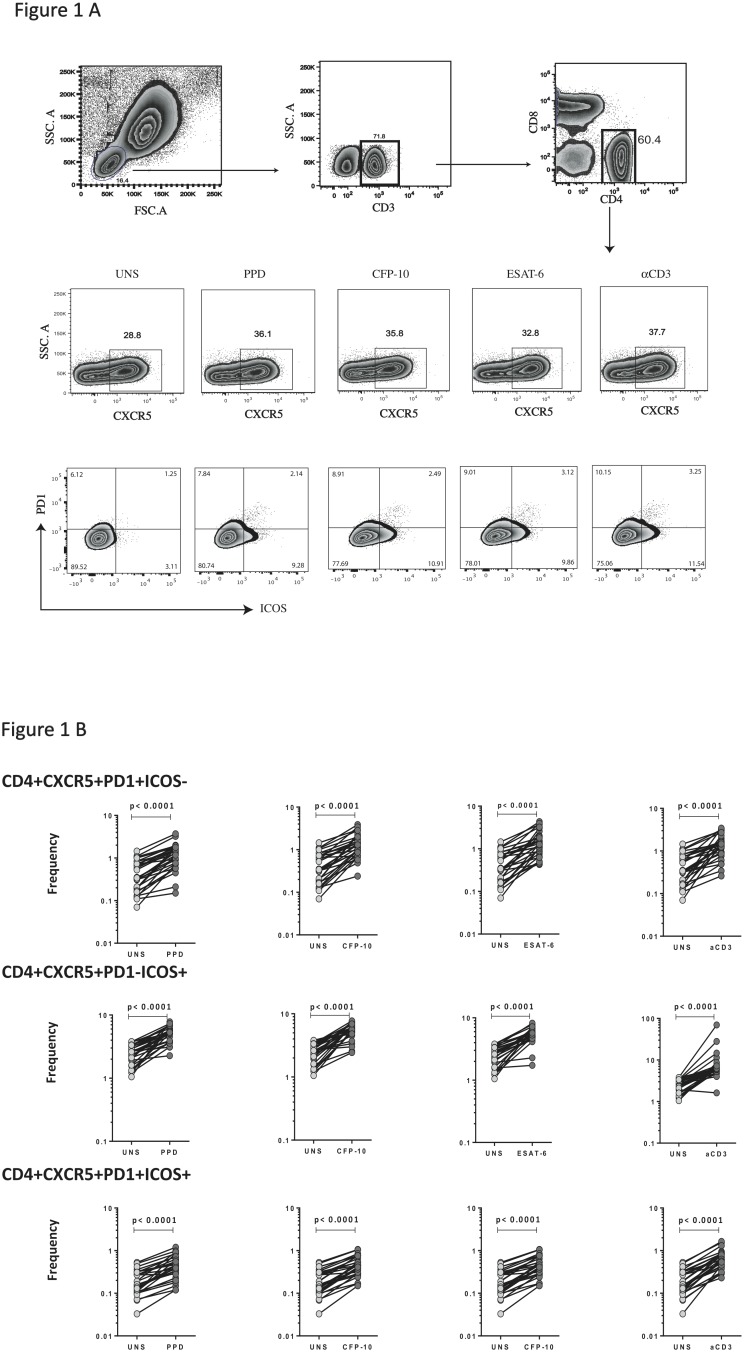
Expansion of Tfh cells subsets in response to TB antigens and anti-CD3 in PTB. Whole blood from PTB individuals was stimulated with media alone (UNS), PPD, CFP-10, ESAT-6 or anti-CD3 for 6 h (Gating strategy for Tfh cell subsets and representative plot shown in A) and (B) the frequencies of CD4^+^ CXCR5^+^ PD-1^+^, ICOS^−^; CD4^+^ CXCR5^+^ PD-1^−^ ICOS^+^ and CD4^+^ CXCR5^+^ PD-1^+^ ICOS^+^ T cells were estimated by flow cytometry. Results are shown as line diagrams with each line representing a single individual. *P* values were calculated using the Wilcoxon signed rank test.

To determine whether Tfh cells are regulated by TB antigens in PTB, we used flow cytometry to first estimate the frequencies of CD4^+^ CXCR5^+^ PD-1^+^ ICOS^−^; CD4^+^ CXCR5^+^ PD-1^−^ ICOS^+^ or CD4^+^ CXCR5^+^ PD-1^+^ ICOS^+^ T cells at baseline or following stimulation with TB antigens or anti-CD3. The gating strategy for Tfh cells is shown in Figure 1A. As shown in Figure 1B, stimulation with PPD, CFP-10, ESAT-6 and anti-CD3 all resulted in significantly increased frequencies (p<0.0001 for all comparisons) of CD4^+^ CXCR5^+^ PD-1^+^ ICOS^−^ T cells, CD4^+^ CXCR5^+^ PD-1^−^ ICOS^+^ T cells and CD4^+^ CXCR5^+^ PD-1^+^ ICOS^+^ T cells from PTB individuals in vitro. Tfh subsets from LTB individuals also increased in frequency in response to TB antigen and polyclonal stimulation (data not shown). Thus, positivity for PD- 1 and/or ICOS define Tfh cells that are capable of responding to TB antigen or polyclonal stimulation in vitro in PTB and LTB individuals.

### Diminished spontaneous as well as TB - antigen induced frequencies of Tfh cell subsets in PTB

**Figure 2:**
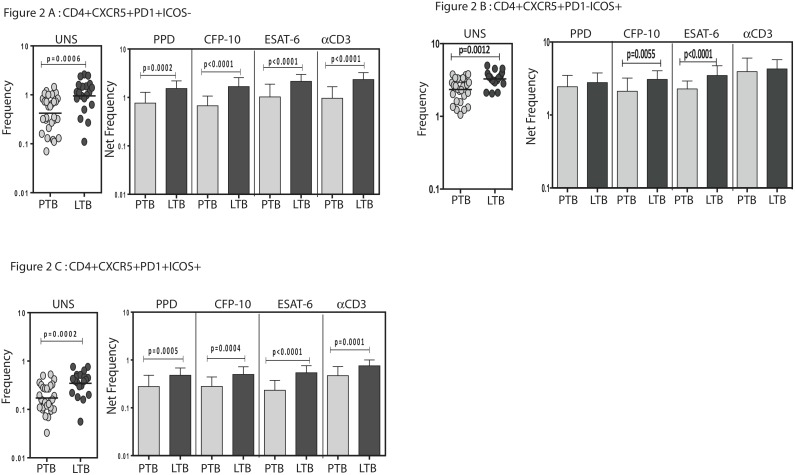
Diminished spontaneous and antigen - specific frequencies of Tfh subsets in PTB and LTB. Whole blood from PTB and LTB individuals was stimulated with PPD, CFP-10, ESAT-6 and anti-CD3 for 6 h, and the frequencies of (A) CD4^+^ CXCR5^+^ PD-1^+^ ICOS^−^; (B) CD4^+^ CXCR5^+^ PD-1^−^ ICOS^+^ and (C) CD4^+^ CXCR5^+^ PD-1^+^ ICOS^+^ T cells were estimated by flow cytometry. Spontaneous frequencies are shown as scatter plots and stimulated frequencies as bar graphs. The bars represent geometric means and 95% confidence intervals. Spontaneous frequencies are shown as raw frequencies and the stimulated frequencies are shown as net frequencies with the spontaneous frequency subtracted. *P* values were calculated using the Mann-Whitney U test.

To compare the baseline (ex vivo) as well as antigen - induced frequencies of Tfh cell subets in PTB and LTB individuals, we measured the frequencies of Tfh cell subsets following antigen stimulation. As shown in Figure 2A, PTB individuals exhibited significantly lower frequencies of both unstimulated as well as TB - antigen and anti-CD3-stimulated CD4^+^ CXCR5^+^ PD-1^+^ ICOS^−^ T cells in comparison to LTB individuals. Similarly, PTB individuals exhibited significantly lower frequencies of unstimulated and TB - antigen (but not anti-CD3) stimulated CD4^+^ CXCR5^+^ PD-1^−^ ICOS^+^ T cells in comparison to LTB individuals (Figure 2B). Finally, PTB individuals exhibited significantly lower frequencies of both unstimulated as well as TB - antigen and anti-CD3- stimulated CD4^+^ CXCR5^+^ PD-1^+^ ICOS^+^ T cells in comparison to LTB individuals (Figure 2C). Thus, active pulmonary TB appears to be characterized by an impaired induction of spontaneous and TB - antigen induced Tfh subsets.

### Diminished TB - antigen induced frequencies of IL-21 expressing Tfh cells in PTB

**Figure 3:**
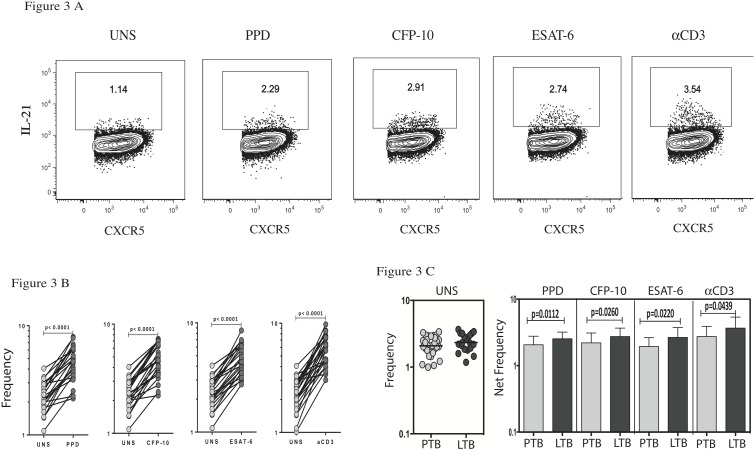
Diminished antigen - specific frequencies of IL-21 expressing Tfh cells in PTB. (A) Whole blood from PTB and LTB individuals was stimulated with PPD, CFP-10, ESAT-6 and anti-CD3 for 6 h, and the frequencies of CD4^+^ CXCR5^+^ IL-21^+^ T cells were estimated by flow cytometry. A representative dot plot is shown. (B) TB antigen- and anti-CD3-induced expansion of IL-21 expressing Tfh cells in PTB. (C) The spontaneous and TB - antigen stimulated frequencies of IL-21 expressing Tfh cells in PTB and LTB individuals. P values were calculated using either Wilcoxon signed rank test or Mann-Whitney U test.

Since IL-21 expression is one hallmark of Tfh cells [3], we sought to determine the role of IL-21 expressing Tfh cells in TB and examined the baseline as well as antigen - induced frequencies of IL-21 expressing Tfh cells in PTB and LTB. As shown in Figure 3A, we were able to detect IL-21 expressing CXCR5^+^ CD4^+^ T cells in the circulation of PTB individuals. We then observed that stimulation with PPD, CFP-10, ESAT-6 and anti-CD3 all resulted in significant increase in the frequency of CD4^+^ CXCR5^+^ IL-21^+^ T cells in PTB individuals (Figure 3B). Next, as shown in Figure 3C, CFP-10, ESAT-6 and anti-CD3 induced frequencies of IL-21 expressing Tfh cells was significantly decreased in PTB compared to LTB individuals. Thus, PTB is characterized by a diminished frequencies of IL-21 expressing Tfh cells in the periphery, indicating diminished function of Tfh cells in PTB.

### Diminished spontaneous frequencies of Tfh cells subsets are associated with diminished circulating IL-21 levels in PTB

**Figure 4:**
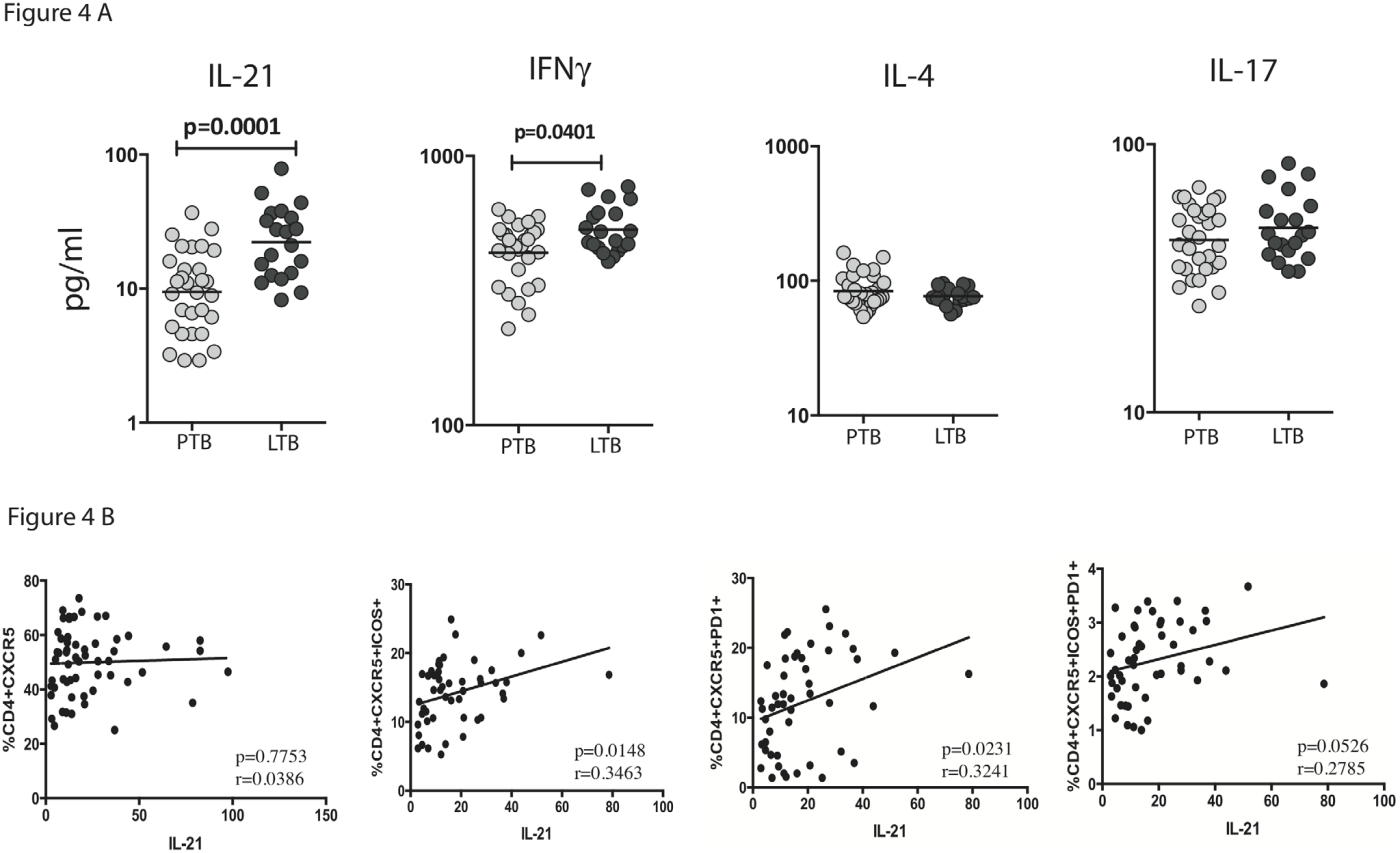
Diminished circulating IL-21 levels in PTB is associated with decreased spontaneous frequencies of Tfh subsets. (A) The circulating levels of Tfh associated cytokines - IL-21, IFNγ, IL-4 and IL-17 were measured by ELISA in PTB and LTB individuals. Results are shown as scatter plots with each circle representing a single individual. *P* values were calculated using the Mann-Whitney U test. (B) The correlation between circulating IL-21 levels and Tfh subsets in shown as scatter plots in PTB individuals. *P* values were calculated using Spearman rank correlation.

Since circulating Tfh cells are also known to produce - IFNγ, IL-4 and IL-17 in addition to IL-21 [12], we determined the circulating levels of these cytokines in PTB and LTB individuals. As shown in Figure 4A, PTB individuals exhibited significantly lower plasma levels of IL-21 and IFNγ but not IL-4 or IL-17 in comparison to LTB individuals. Interestingly, when we examined the relationship between the spontaneous frequencies of Tfh cell subsets and the circulating levels of IL-21, we observed a significant positive correlation between the frequencies of CD4^+^ CXCR5^+^ ICOS^+^ T cells and CD4^+^ CXCR5^+^ PD-1^+^ T cells and plasma levels of IL-21 (Figure 4B). No significant correlation was observed between the spontaneous frequencies of Tfh cell subsets and plasma levels of IFNγ (data not shown). Thus, diminished spontaneous frequencies of Tfh cells subsets is significantly and directly associated with diminished IL-21 levels in PTB.

### PTB is not associated with alterations in memory B cells or plasma cell frequencies

**Figure 5:**
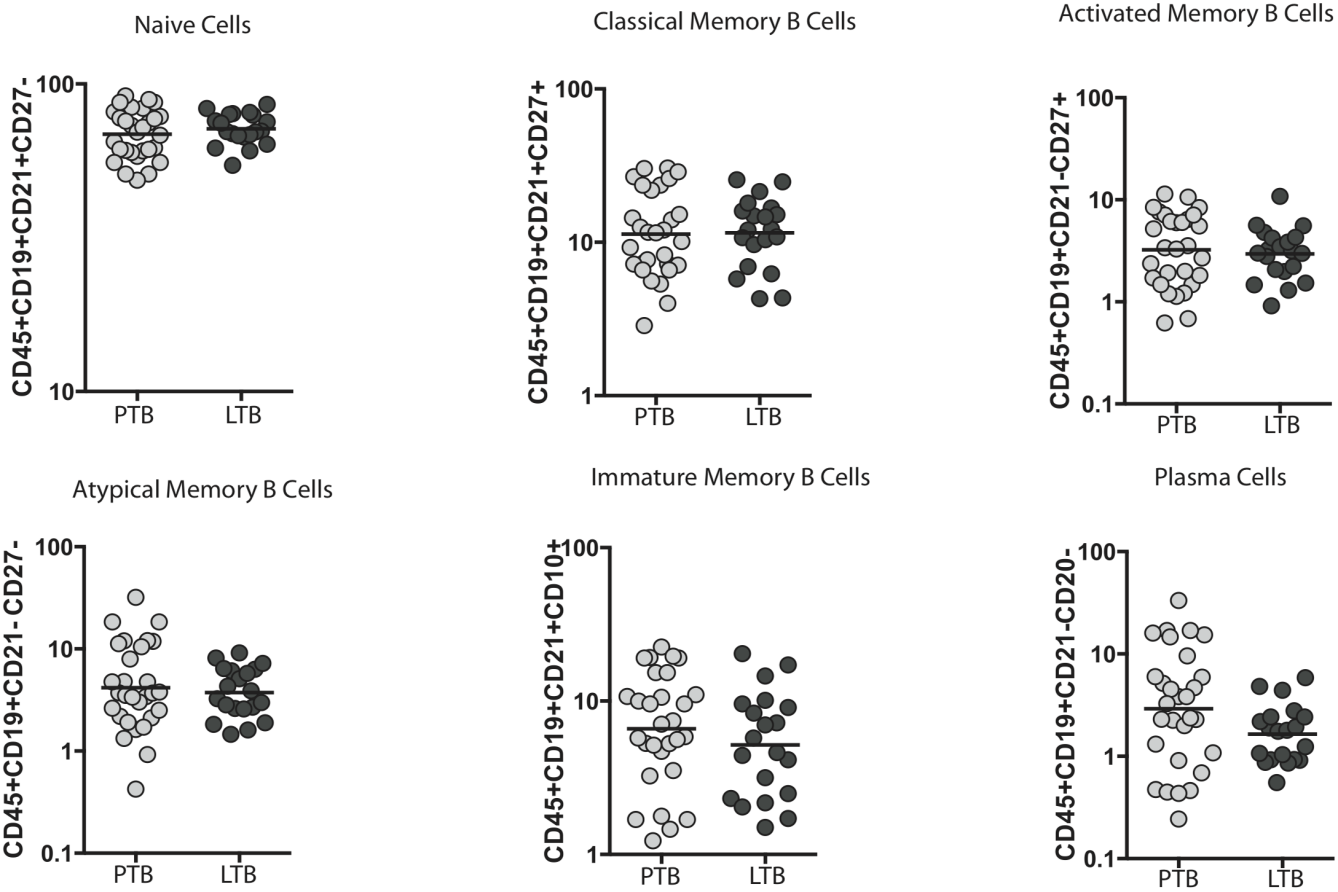
The distribution of B cells subsets in TB infection and disease. Ex vivo phenotyping of B cell subsets was performed on PTB and LTB individuals by flow cytometry. B cells were classifed as naïve B cells (CD45^+^CD19^+^CD21^+^CD27^−^); classical memory B cells (CD45^+^CD19^+^CD21^+^CD27^+^); activated memory B cells (CD45^+^CD19^+^CD21^−^CD27^+^); atypical memory B cells (CD45^+^CD19^+^CD21^−^CD27^−^); immature B cells (CD45^+^CD19^+^CD21^+^CD10^+^) and plasma cells (CD45^+^CD19^+^CD21^−^CD20^−^). *P* values were calculated using Mann-Whitney U test.

Since circulating Tfh cells are known to promote activated or classical memory B cell and plasma cell formation [12], we determined if the alteration in Tfh cell subset frequencies would translate to altered frequencies of activated or classical memory B cell and plasma cell subsets. We therefore measured the frequency of naive B cells, classical memory B cells, activated memory B cells, atypical memory B cells, immature B cells and plasma cells immediately ex vivo in PTB or LTB individuals. As shown in Figure 5, PTB individuals exhibited no significant difference in any of the above subsets of B cells in comparison to LTB individuals.

### IL-10, CTLA-4 and PD-L1 regulate the antigen - stimulated frequencies of Tfh cell subsets in PTB

**Figure 6:**
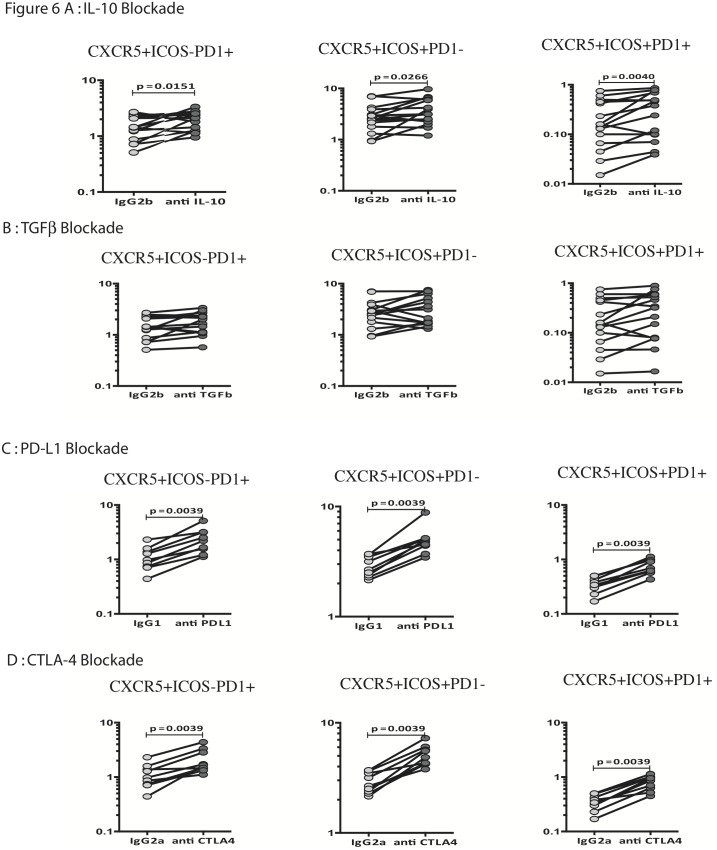
Blockade of IL-10, CTLA-4 and PD-L1 but not TGFβ significantly enhances Tfh cell subset frequencies in PTB. Whole blood from PTB individuals was stimulated with PPD in the presence of anti-IL-10 Ab (A) or anti-TGFβ Ab (B) or isotype control antibodies and in the presence of anti- CTLA-Ig (C) or anti-PD-L1 (D) or isotype control antibody for 18 h and the frequencies of CD4^+^ CXCR5^+^ PD-1^+^, ICOS^−^; CD4^+^ CXCR5^+^ PD-1^−^ ICOS^+^ and CD4^+^ CXCR5^+^ PD-1^+^ ICOS^+^ T cells were estimated by flow cytometry. Results are shown as line diagrams with each line representing a single individual. *P* values were calculated using the Wilcoxon signed rank test.

Since IL-10 and TGFβ are known regulators of T cell function, we determined the role of these regulatory cytokines in the diminished frequencies of Tfh cell subsets in PTB. Thus, we stimulated whole blood from PTB individuals with PPD in the presence of neutralizing antibodies to IL-10 or TGFβ and measured the frequencies of Tfh cell subsets. As shown in Figure 6A, blockade of IL-10 resulted in a significant increase in the frequencies of PPD-stimulated CD4^+^ CXCR5^+^ PD-1^+^ ICOS^−^ or CD4^+^ CXCR5^+^ PD-1^−^ ICOS or CD4^+^ CXCR5^+^ PD-1^+^ ICOS^+^ T cells. In contrast, as shown in Figure 6B, TGFβ blockade had no significant effect on the frequencies of the Tfh cell subsets.

Because CLTA-4 and PD-1 are also known regulators of T cell function, we also wanted to determine the role of these receptors in the diminished frequencies of Tfh cell subsets in PTB. We therefore, stimulated whole blood from PTB individuals with PPD in the presence of blocking antibodies to CTLA-4 or PD-L1 and measured the frequencies of Tfh cell subsets. As shown in Figure 6C, blockade of CTLA-4 signaling resulted in significantly higher frequencies of CD4^+^ CXCR5^+^ PD-1^+^ ICOS^−^ or CD4^+^ CXCR5^+^ PD-1^−^, ICOS^+^ or CD4^+^, CXCR5^+^ PD-1^+^ ICOS^+^ T cells in PTB individuals. Similarly, as shown in Figure 6D, blockade of PD-1 signaling also resulted in significantly higher frequencies of Tfh cell subsets. Interestingly, IL-10, CTLA-4 and PD-1 did not significantly alter the frequency of Tfh cell subsets in LTB individuals (data not shown). Thus, the regulatory cytokine - IL-10 as well as the co-inhibitory molecults - CTLA-4 and PD-1 appear to function to limit the frequencies of Tfh cell subsets in PTB.

## Discussion

Infection with Mtb can lead to various outcomes that range from active or chronic pulmonary disease, extra-pulmonary TB and latent TB, that occurs when the initial infection is controlled but not completely eliminated [13]. While a number of host immune mechanisms have been described to play a role in the diverging clinical manifestations of TB infection and disease, the immune mechanisms that contribute directly to disease pathogenesis are still incompletely understood [14]. Tfh cells are a subset of CD4^+^ T cells that are indispensable for the generation and maintenance of humoral immunity [3]. It has recently been demonstrated that CXCR5^+^ T cell accumulate within ectopic lymphoid structures associated with TB granulomas in humans, non-human primates and mice [11]. These lymphoid follicles appear to be important for proper localization of T cells in the granulomas, for the optimal activation of macrophages and for protection against TB disease [11]. Thus, while Tfh cells located within the granulomas are clearly important in the immune response to TB, the role of circulating Tfh cells in human TB infection and disease remains unexplored.

The distribution of Tfh cells in TB infection and disease was studied by classifying them into 3 subsets. We first attempted to infer the function of these Tfh subsets expressing different combination of surface markers by examining their response to MTB antigens in those with LTB and those with active PTB. Our data clearly reveals that both PD-1 and ICOS either separately or together mark Tfh cells with similar properties in terms of antigenic responsiveness. Subsequently, the examination of frequencies of these Tfh subsets revealed a significant reduction in the frequencies of these subsets in those with PTB. This study demonstrates for the first time, we believe, that there are decreased frequencies of Tfh cells in tuberculosis, data that are in contrast to data from HIV and other viral infections where Tfh frequencies [15], [16] are increased. Thus, PTB appears to be selectively associated with TB - antigen specific deficiency in Tfh cells expressing either ICOS and/or PD-1. One of the major hallmarks of Tfh cells is their ability to produce a variety of cytokines - most notably IL-21 [3]. Our examination of the IL-21 expressing Tfh cells in PTB reveals that the diminished frequency of Tfh cells in TB disease translated to diminished function of these cells. In addition, we also observed significantly diminished systemic levels of IL-21 in PTB and a significant positive correlation between Tfh cell subset frequencies and IL-21 levels in these individuals suggesting that most of the IL-21 in TB infection and disease is probably derived from circulating Tfh cells. Since there are increasing reports of Tfh cells producing other cytokines including IL-4, IFNγ and IL-17 [12], we examined the levels of these cytokines and found lower levels of circulating IFNγ in PTB. Thus, we demonstrate that the dysregulated expression of Tfh cell subsets is actually associated with functional impairment of these cells in the circulation of PTB individuals. It is possible that the diminished frequencies in the blood of PTB individuals is a reflection of increased migration to the tissues - lungs or mediastinal lymph nodes. However, since circulating Tfh cells have been shown to consistently exhibit additional functions compared to tissue resident Tfh cells, our data hold importance in terms of potential impact on pathogenesis of TB disease. However, we have not examined the frequencies of these cells or that of their hallmark cytokines in healthy control individuals (without latent infection) and therefore need to perform these studies in the future to elucidate the role of these cells and cytokines in TB infection.

Another hallmark of Tfh cells is their ability to provide B cell help [6]. Although the role of B cells in TB is not clearly understood, it is known that B cell deficient mice appear more susceptible to TB [17]. Studies of human and non human primate TB granulomas have identified B cell follicle structures that might contribute to the immune response to TB [11], [18]. While conventional Tfh cells are known to promote all aspects of B cell function, circulating Tfh cells are known only to specifically promote activated and classical memory B cell and plasma cell formation [19]. However, we did not observe any significant compromise in memory B cell formation in PTB nor were there any perturbations in frequencies of other B cell subsets. Again, we have not estimated the function of these cells directly nor have we estimated the frequency of these cells in healthy control indviduals (without latent infection).

Although it is known that numerous cytokines can promote Tfh formation, much less is known about the factors that restrain this process. It has been reported that Tfh cell formation can be suppressed by several cytokines, including IL-10 and TGFβ [4]. Indeed, we also detected increased levels of IL-10 in circulation in PTB individuals. Coupled with data on the blockade of IL-10 in PTB individuals, it appears that IL-10 does indeed play an important role in the modulation of Tfh cells in active PTB. On the other hand, TGFβ appears to play a negligible role in the regulation of Tfh cells in active TB. Although PD-1 is highly expressed in Tfh cells, little is known about the role of PD-1 in Tfh cell development and function. Studies in mice deficient in PD-1 or its ligands report increased numbers of Tfh cells, suggesting that PD-1/PD-L1 interactions downregulate Tfh generation and/or differentiation [20]. While CTLA-4 is a potent inhibitor of effector T cell differentiation and function [21], its role in regulating Tfh cell expansion is not known. Interestingly, our study reveals an important role for both PD-1 and CTLA-4 signaling in the down modulation of Tfh cell expansion in pulmonary TB since blockade of either of these pathways significantly restored the TB - antigen induced expansion of Tfh cell subsets in PTB individuals. These data provide novel insights into the role of PD-1 and CTLA-4 on the regulation of Tfh cells in a human infectio and suggest that imporant new roles for these molecules apart from their effect on T effector cells.

In summary, our data on the Tfh cell distribution and function in pulmonary tuberculosis suggest that compromised Tfh cell numbers and function are a prominent feature of active disease. Although our study was not designed to decipher cause and effect mechanisms of Tfh association with active TB, it nevertheless implicates an important role for this poorly explored subset of CD4^+^ T cells in active TB. Our study also provides the first comprehensive analysis of the distribution of B cell subsets in pulmonary TB and reveals that compromised B cell subset distribution is not a feature of active TB disease. Our data also suggest that adjunct immuno-modulation in the form of co-inhibitory receptor blockade (or immune checkpoint blockade using CTLA-4 and PD-1 antagonists) could potentially also enhance the protective immune responses of susceptible individuals.
